# Safety of live attenuated influenza vaccine (LAIV) in children with moderate to severe asthma

**DOI:** 10.1016/j.jaci.2019.12.010

**Published:** 2020-04

**Authors:** Paul J. Turner, Louise Fleming, Sejal Saglani, Jo Southern, Nick J. Andrews, Elizabeth Miller, SNIFFLE-4 Study Investigators, Alexandra Adams, Christine Doyle, Michel Erlewyn-Lajeunesse, Katy Fidler, Louise Fleming, Atul Gupta, Stephen M. Hughes, Andrew Ives, Nicola Jay, Sonal Kansra, Louise Michaelis, Samantha Moss, Clare Murray, Prasad Nagakumar, Graham Roberts, Sejal Saglani, Paul Seddon, Ian Sinha, Gary Stiefel, Huw M. Thomas, Paul J. Turner

**Affiliations:** a Section of Inflammation, Repair & Development, National Heart & Lung Institute, Imperial College London, London, United Kingdom; b Immunisation, Hepatitis and Blood Safety Department, Public Health England, London, United Kingdom

**Keywords:** Asthma, children, immunization, influenza, live attenuated influenza vaccine, ACQ, Asthma Control Questionnaire, ACT, Asthma Control Test, AE, Adverse event, (c)ACT, (Children’s) Asthma Control Test, ICS, Inhaled corticosteroid, IIV, Injected influenza vaccine, LAIV, Live attenuated influenza vaccine, TRACK, Test for Respiratory and Asthma Control for Kids

## Abstract

**Background:**

Live attenuated influenza vaccine (LAIV) is recommended for annual influenza vaccination in children from age 2 years. However, some guidelines recommend against its use in children with asthma or recurrent wheeze due to concerns over its potential to induce wheezing.

**Objective:**

We sought to assess the safety of LAIV in children with moderate to severe asthma, and in preschool children with recurrent wheeze.

**Methods:**

Prospective, multicenter, open-label, phase IV intervention study in 14 specialist UK clinics. LAIV was administered under medical supervision, with follow-up of asthma symptoms 72 hours and 4 weeks late, using validated questionnaires.

**Results:**

A total of 478 young people (median, 9.3; range, 2-18 years) with physician-diagnosed asthma or recurrent wheeze were recruited, including 208 (44%) prescribed high-dose inhaled corticosteroids and 122 (31%) with severe asthma. There was no significant change in asthma symptoms in the 4 weeks after administration (median change, 0; *P* = .26, McNemar test), with no impact of level of baseline asthma control/symptoms in predicting either a worsening of asthma or exacerbation after LAIV using a regression model. A total of 47 subjects (14.7%; 95% CI, 11%-19.1%) reported a severe asthma exacerbation in the 4 weeks after immunization, requiring a short course of systemic corticosteroids; in 4 cases, this occurred within 72 hours of vaccination. No association with asthma severity, baseline lung function, or asthma control was identified.

**Conclusions:**

LAIV appears to be well tolerated in the vast majority of children with asthma or recurrent wheeze, including those whose asthma is categorized as severe or poorly controlled.

Annual influenza vaccination for children older than 6 months has been recommended by the Centers for Disease Control and Prevention and its Advisory Committee on Immunization Practices since 2010.^1^ Children are the main spreaders of influenza infection; vaccinating this age group is considered to be the most effective method for interrupting transmission and achieving disease control.^2^ The Advisory Committee on Immunization Practices highlights a number of groups at a higher risk for severe complications from influenza, including children aged 6 through 59 months, and adults and children with chronic pulmonary disease (including asthma).^3^ One option for immunization is the live attenuated influenza vaccine (LAIV), an intranasally administered vaccine that is popular among both parents and health care professionals due to the noninvasive route of administration.

Annual influenza vaccination of all children was introduced into the National Immunization schedule in the United Kingdom in 2013. The program uses LAIV and in a staged implementation is currently targeting all children from age 2 to 11 years, as well as children considered to be at a higher risk of influenza infection (including those with asthma and recurrent wheeze).^4^ Surveillance data have demonstrated the effectiveness of the program,^5^ and the safety of LAIV in the general population has been demonstrated in a number of published studies.^6^

However, the Advisory Committee on Immunization Practices recommends against LAIV in preschool children with history of at least 1 wheezing episode in the past 12 months, and for the vaccine to be used with caution in older children (≥5 years) with asthma,^3^ due to limited evidence from 2 clinical trials that LAIV may induce wheezing in younger children.^7^^,^^8^ These concerns have not been reproduced in other studies^9^, ^10^, ^11^, ^12^ nor through postmarketing surveillance.^13^, ^14^, ^15^, ^16^, ^17^

In the UK setting, where up to 9% of children have a diagnosis of asthma,^18^ the SNIFFLE-2 study provided evidence for the safety of LAIV in children with mild to moderate asthma. However, there are insufficient data in children requiring higher doses of inhaled corticosteroids (ICSs) or with “difficult” asthma to make any recommendations in these cohorts.^19^ Elsewhere, there has been significant heterogeneity in defining poorly controlled or “difficult” asthma in postmarketing surveillance.^16^^,^^17^ We therefore sought to assess the safety of LAIV in children with moderate to severe asthma in a multicenter, prospective interventional study.

## Methods

This was a phase IV open-label study of LAIV in young people with asthma during the 2016/2017 influenza season across 14 specialist pediatric asthma clinics in the United Kingdom. Eligible participants were aged 2 to 18 years, with a current physician diagnosis of asthma and prescribed regular ICS. Preschool-age children (between 2 and 5 years) were also eligible if they had experienced 2 or more exacerbations of wheezing in the previous year requiring oral steroids or hospitalization. A number of different (but established) definitions were used to assign severity, to reflect differences in national and international practice (outlined in Table I^20^, ^21^, ^22^). Children requiring oral steroids or receiving biologics such as omalizumab were not excluded. Participants were excluded if they had previously required invasive ventilation because of a respiratory illness in the preceding 2 years or had a contraindication to LAIV (egg allergy notwithstanding). Vaccination was deferred for acute febrile illness. We did not exclude children with acute wheeze, unless occurring within the previous 72 hours in the context of a new acute exacerbation.

**Table I:** Baseline demographic characteristics of the cohort by severity definition

Characteristic	Age (y)			Overall cohort (n = 478)
	2-4 (n = 85)	5-11 (n = 235)	12+ (n = 158)	
Sex: male	52 (61)	151 (64)	85 (54)	288 (60)
Baseline lung function				
FEV_1_ (% predicted), median (IQR)	NA	91 (78-102)	92 (80-99)	NA
		(n = 138)	(n = 122)	
PEFR (% predicted), median (IQR)	NA	87 (75-101)	91 (85-100)	NA
		(n = 58)	(n = 48)	
Baseline asthma control score, median (IQR)				
TRACK	55 (35-65)	NA	NA	NA
(c)ACT	NA	19 (15-22)	19 (16-22)	
ACQ	NA	6 (2-12)	6 (3-11)	
Previous influenza vaccination	41 (48)	186 (79)	120 (76)	332 (69)
Previous LAIV	24 (28)	134 (57)	64 (41)	222 (46)
Asthma severity				
“Persistent poor control”^20^:				
Age 2-4 y: ICS >200 μg + LTRA or ≥2 exacerbations in past year requiring oral steroids/hospitalization	68 (80)	88 (37)	73 (46)	229 (48)
Age 5-11 y: ICS ≥ 400 μg/d				
Age 12+y: ICS ≥ 800 μg/d				
High-dose ICSs, n (%)				
Age 2-4 y: ICS ≥ 400 μg/d	48 (57)	63 (27)	97 (61)	208 (44)
Age 5+ y: ICS ≥ 800 μg/d				
Severe asthma (according to ATS/ERS guidelines)^22^				
High-dose ICS AND at least 1 of:	NA	51 (22)	71 (45)	122 (31∗)
ACT score <20				
≥2 courses of systemic steroids in the past year				
Serious exacerbation (hospital admission in last year)				
FEV_1_ <80%				
“Difficult asthma”: persistence of symptoms despite treatment with high-dose therapies^21^	54 (64)	46 (20)	42 (27)	142 (30)

*ATS*, American Thoracic Society; *ERS*, European Respiratory Society; *IQR*, interquartile range; *LTRA*, leukotriene receptor antagonist; *NA*, not applicable/available; *PEFR*, peak expiratory flow rate.

Values are n (%) unless otherwise indicated.

∗ Excluding study participants <5 years.

The study was approved by the West Midlands-Edgbaston Research Ethics Committee (16/WM/0276), and the parent/guardian of each participant gave written informed consent. Children older than 8 years were encouraged to provide assent. The study sponsor was Imperial College Healthcare NHS Trust. This study was registered with ClinicalTrials.gov (NCT02866942) and the EU Clinical Trials Register, EudraCT (2016-002352-24).

### Procedures

Participants underwent clinical evaluation (including vital signs and lung function [FEV_1_ and/or peak expiratory flow rate] where age-appropriate). They (or their parents) were also asked to complete the following age-appropriate validated questionnaires to assess asthma control:

• Age 2 to 4 years: Test for Respiratory and Asthma Control for Kids (TRACK)^23^
• Age 5 to 11 years: Asthma Control Questionnaire (ACQ)^24^ and Children’s Asthma Control Test ((c)ACT),^25^ which assess asthma symptoms/control over the preceding 1 and 4 weeks, respectively;
• Age 12+ years: ACQ and Asthma Control Test (ACT).

Subjects were then administered quadrivalent LAIV (Fluenz-Tetra, produced for the 2016/2017 influenza season) following the method specified on the approved summary of product characteristics. Parents were telephoned at least 72 hours after vaccination to document any adverse events (AEs), and then contacted by either email or telephone 4 weeks later to reassess ACT score and associated symptoms. Vaccine-naive subjects younger than 9 years were offered a second dose of LAIV 4 weeks later, in line with national guidelines.

### Outcomes

The primary outcome was the change in asthma control before and in the 4 weeks after LAIV in young people with asthma/recurrent wheezing, as assessed by age-appropriate questionnaire (TRACK or (c)ACT), and by severity classification. Clinical secondary outcomes were incidence of AEs including severe asthma exacerbation and serious AEs (sAEs) in the 4 weeks after LAIV in study participants. We used the American Thoracic Society/European Respiratory Society definition for a severe asthma exacerbation, namely, (1) use of systemic corticosteroids or an increase from a stable maintenance dose, for at least 3 days; OR (2) unscheduled visit to an emergency department or hospital admission because of asthma, requiring systemic corticosteroids.^26^ AEs were reviewed by an independent data monitoring committee, and causality assigned in conjunction with local study teams.

### Statistical analyses

Analyses were planned prospectively and detailed in a statistical analysis plan. For the primary outcome, the change in TRACK or (c)ACT score pre- and 4 weeks post-LAIV was assessed by McNemar test for paired data. The minimum important difference for the (c)ACT score is around 2 points in younger children^25^ and 3 points in adults,^27^ and 10 points for TRACK.^23^ For the purpose of this analysis, a change in (c)ACT of at least 3 points or 10 points for TRACK was determined to be a clinically relevant change, where this resulted in a change in symptom control from reasonable ((c)ACT score ≥20 or TRACK score ≥80 points) to suboptimal control ((c)ACT score <20 or TRACK score <80 points). Severity was defined as outlined in Table I, and a further definition was included as an additional post hoc analysis to reflect the new definition for difficult asthma adopted by the British Thoracic Society in 2016 and included in the 2019 guideline.^21^ Subgroup analyses were performed according to asthma severity, previous vaccination status, and by age group (2-4, 5-11, 12+ years). For secondary outcomes, the incidence of reactions (immediate and delayed) and significant asthma exacerbation after LAIV were estimated with 2-sided exact 95% CIs. We also developed a regression model to assess for baseline characteristics associated with the occurrence of significant exacerbation. The analysis data set was as treated and with the relevant safety data measured.

Sample size was determined on the primary objective. A total of 720 children (split 50:50 into higher/regular dose of ICS) would give 80% power at a 5% significance level to detect an improvement in 10% versus deterioration in 17.5% as significant. Increasing sample size to 840 would allow for approximately 15% attrition. In reality, recruitment was lower than planned and attrition greater, such that the study was powered to detect only larger differences (eg, with n = 300, 10% vs 22.5%).

## Results

**Fig 1:**
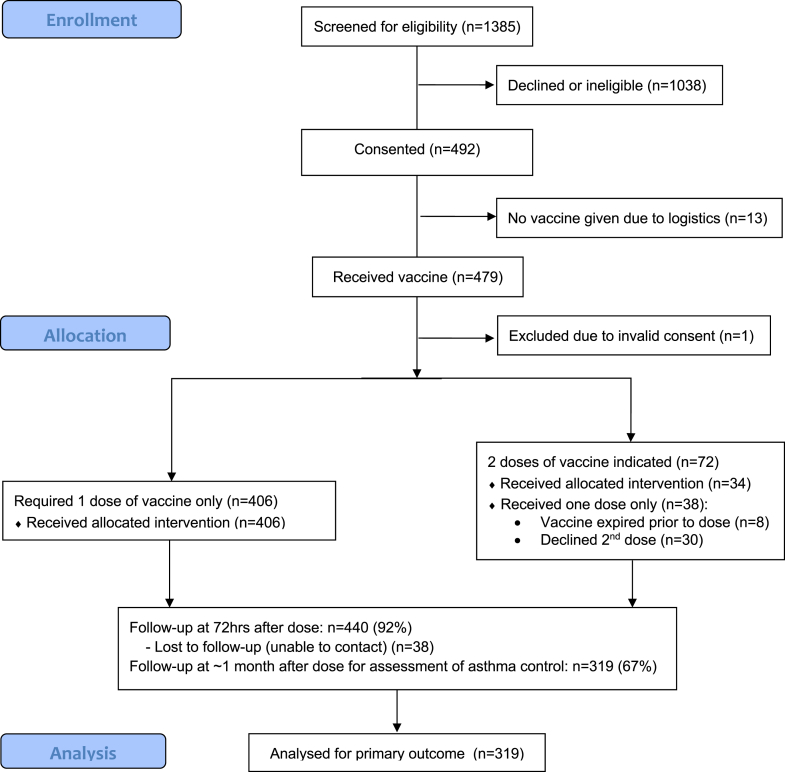
Participant flow diagram. Thirteen children had consent but were not eligible for the study: 6 due to recent antihistamine use (family declined a further study appointment); 5 refused after parental consent had been obtained; 2 with unstable asthma (and who were given IIV instead).

A total of 479 children were recruited and received at least 1 dose of LAIV. One child was excluded because of improper consent, leaving an analysis cohort of 478 participants. The median age of the cohort was 9.3 years (range, 2-18 years) and 288 (60%) were males. A total of 332 (69%) had received influenza vaccination previously, of whom 222 had been given LAIV. All participants had physician-diagnosed asthma or recurrent wheeze (Table I). Coexisting atopic diseases were common: 63% had allergic rhinitis, 48% eczema, and 44% food allergy. A second LAIV dose was administered to 34 children because of no previous vaccination; a further 38 were eligible for a second dose, but did not receive it because of expiry of the vaccine (8 children) or the family declining a second visit (30 children), as shown in the CONSORT diagram (Fig 1).

### Primary outcome: Change in asthma control after LAIV

**Fig 2:**
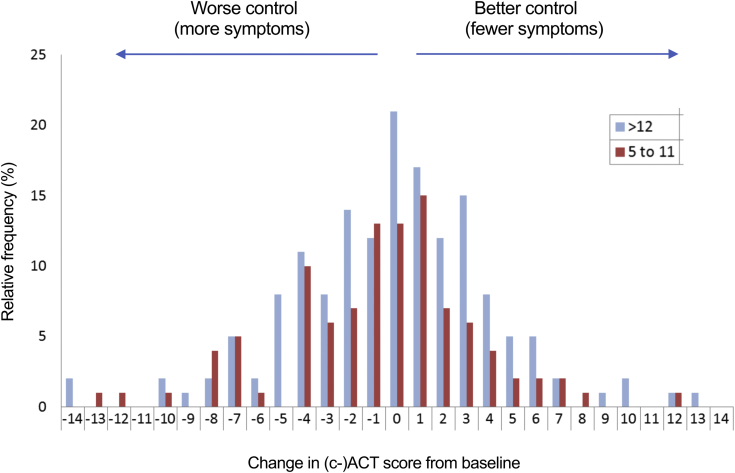
Change in ACT score at 4 weeks post-LAIV, compared with baseline, in children older than 5 years with a history of asthma.

Follow-up data for asthma control were available for 319 of 478 (67%) participants: 71%, 67%, and 65% aged 2 to 4, 5 to 11, and 12+ years, respectively. There were no obvious differences in age, baseline asthma control, and previous vaccination status between those in whom follow-up data were available and those without (see Table E1 in this article’s Online Repository at www.jacionline.org). We did observe fewer responses in teenagers receiving high-dose ICSs, but not in the preschool or 5- to 11-year age group. The change in (c)ACT score for the 4 weeks after LAIV administration in subjects older than 5 years is shown in Fig 2. There was no significant change in (c)ACT score for children 5 years and older (median change, 0; *P* =.18, McNemar test), with 58 (22%) subjects reporting an improvement in asthma control at least equal to the minimum important difference and 70 (27%) reporting a deterioration. There was no significant change in asthma control by age of child (5-11 and 12+ years; *P* = .71 and .08, respectively). Analyzing the data according to severity of asthma, we did not identify any particular severity definition associated with a significant change in asthma control (Table II; see Table E2 in this article’s Online Repository at www.jacionline.org), although subjects aged 12+ years without “difficult asthma” paradoxically reported a mild worsening in ACT (although this analysis might have been affected by regression to the mean). Previous vaccination status did not affect change in asthma control (*P* = .36).

**Table II:** Change in symptom control ≥minimum important difference for study population (and by severity definition) where this resulted in a change in symptom control from well controlled to suboptimal (or vice versa)

Population	n	Improved	No change	Worse	McNemar *P* value
Complete cohort					
All ages	319	28 (9)	254 (80)	37 (12)	.26
2-4 y	60	3 (5)	49 (82)	8 (13)	.13
5+ y	259	25 (10)	205 (79)	29 (11)	.59
“Persistent poor control”					
All ages	147	12 (8)	118 (80)	17 (12)	.35
2-4 y	45	3 (7)	37 (82)	5 (11)	.48
5+ y	102	9 (9)	81 (79)	12 (12)	.51
High-dose ICS					
All ages	127	8 (6)	106 (83)	13 (10)	.26
2-4 y	33	2 (6)	29 (88)	2 (6)	1.00
5+ y	94	6 (6)	77 (82)	11 (12)	.23
“Difficult asthma”					
All ages	90	7 (8)	79 (88)	4 (4)	.37∗
2-4 y	37	3 (8)	30 (81)	4 (11)	.71
5+ y	53	4 (8)	49 (92)	0 (0)	.05∗

Values are n (%).

∗ This severity definition is partially dependent on the (c)ACT score, so this *P* value may be influenced by regression to the mean.

**Fig 3:**
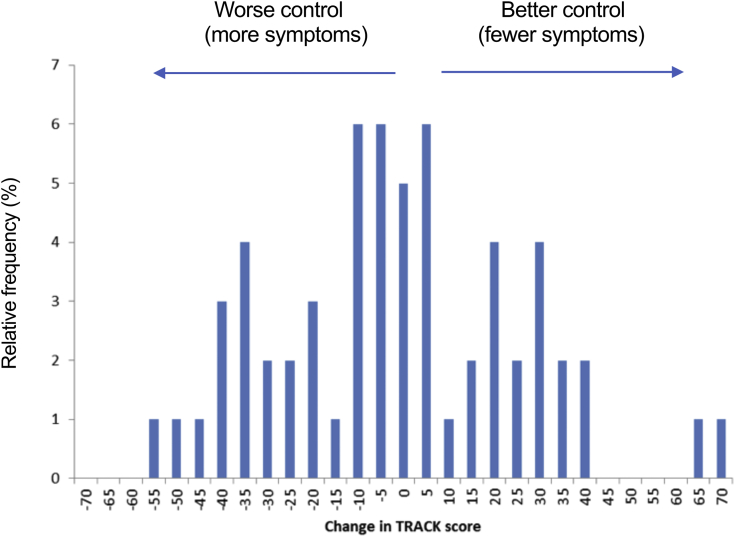
Change in TRACK score at 4 weeks post-LAIV, compared with baseline, in children aged 2 to 4 years with a history of recurrent wheeze.

Children younger than 5 years were initially analyzed separately, because of the use of a different tool (TRACK) to evaluate any potential change in respiratory symptoms. There was no significant change in TRACK score after LAIV overall (Fig 3, *P* = .54, McNemar test), nor by severity subgroup.

Combining all age groups, Table II reports the number of patients with a significant change in symptom control across all ages for the analysis of primary outcome, and by severity classification. Overall, the proportion of subjects with a significant deterioration after LAIV (37 of 319 [11.6%]; 95% CI, 8.3%-15.6%) was similar to the proportion of subjects reporting improved control (28 of 319 [8.8%]; *P* = .26); thus, these data do not provide evidence that any deterioration in symptom control was due to receipt of LAIV.

### AEs after immunization

Nine AEs were reported (in 9 individuals) within 2 hours of vaccination (see Table E3 in this article’s Online Repository at www.jacionline.org). Six were not consistent with a potential IgE-mediated allergic response (as defined by international consensus criteria) thought to be related to vaccine. Thus, the rate of acute AEs after immunization was 3 of 478 or 0.63% (95% CI, 0.13%-1.82%), which is in line with the reported rate of AEs described in the literature for the normal population.^7^

Follow-up to 72 hours was completed for 440 (92%) participants. Two hundred five delayed AEs were reported by parents in 150 children. After excluding 11 AEs that were deemed unlikely to have been vaccine-related, a total of 139 children experienced delayed events (occurring between 2 and 72 hours after vaccine administration), described in Table III. Twenty-eight of 440 children (6.4%; 95% CI for population, 4.3%-9.1%) experienced parent-reported wheezing within 72 hours, of whom 4 were prescribed a short course of systemic corticosteroids. More delayed AEs were reported in the younger ages (which may reflect an increased rate of viral infections in this age group), but otherwise the occurrence of delayed AEs was not associated with any particular baseline characteristic, including baseline ACT/ACQ score or lung function (see Table E4 in this article’s Online Repository at www.jacionline.org).

**Table III:** Delayed AEs occurring up to 72 hours after LAIV immunization, as reported by parents from 440 children with 72-hour follow-up

AE	No. of children	Rate (95% CI) in this cohort
Upper respiratory tract		
Upper respiratory tract (any)	55	12.5% (9.6%-16.0%)
Isolated symptoms only, <24-h duration	23	5.2% (3.3%-7.7%)
Isolated symptoms only, >24-h duration	31	7.1% (4.8%-9.9%)
Nasal symptoms with ocular involvement	1	0.2% (0.1%-1.3%)
Lower respiratory tract		
Lower respiratory tract (any)	56	12.7% (9.8%-16.2%)
Parent-reported wheeze	28	6.4% (4.3%-9.1%)
Constitutional		
Any	58	13.2% (10.2%-16.7%)
Fever <24 h	10	2.3% (1.1%-4.1%)
Fever >24 h	7	1.6% (0.6%-3.3%)
Other: lethargy, headache, dizziness, myalgia	42	9.6% (7.0%-12.7%)
Dermatological		
Flare in eczema	3	0.7% (0.1%-2.0%)
Nonspecific rash, no response to antihistamine	5	1.1% (0.4%-2.6%)
Abdominal symptoms		
Vomiting, nausea, abdominal pain	9	2.1% (0.9%-3.9%)
Loose stools	2	0.5% (0.1%-1.6%)
Ear-nose-throat		
Mild nose bleed	3	0.7% (0.1%-2.0%)
Sore throat	4	0.9% (0.3%-2.3%)
Ocular		
Itch, redness	1	0.2% (0.01%-1.3%)
Neurological		
Any	0	0% (0.0%-0.8%)
Cardiovascular		
Any	0	0% (0.0%-0.8%)

### Asthma exacerbation after 72 hours and SAEs

There were 4 SAEs during the study, 2 of which were considered potentially attributable to LAIV (details are provided in Table E5 in this article’s Online Repository at www.jacionline.org). In total, 47 of 319 subjects (9 of 60 children aged 2-4 years, and 22 of 157 and 16 of 102 subjects aged 5-11 and 12+ years, respectively) reported a severe asthma exacerbation in the 4 weeks after immunization, giving a rate of 14.7% (95% CI, 11.0-19.1). We developed a logistic regression model to try and predict the risk of exacerbation on the basis of asthma control and lung function before LAIV, for each age group. No association between baseline asthma control and odds of exacerbation was identified for the 2- to 4-year and the 5- to 11-year age groups (*P* = .69 and .65, respectively). For subjects aged 12+ years, there was no association with baseline lung function or ACQ score after adjusting for ACT score (*P* = .17 and .66, respectively). Baseline ACT score was more strongly associated with exacerbation, with an odds ratio of 0.29 per 5 points (95% CI, 0.14-0.59; *P* = .001); however, the area-under-receiver-operating-characteristic curve was 0.796, which implies that the model was not highly predictive.

## Discussion

We did not find any evidence that administration of LAIV in young people with severe or “difficult” asthma resulted in an adverse safety signal, including in preschool-age children with severe wheezing. Although 15% of participants reported an asthma exacerbation in the 4 weeks after LAIV, this was not associated with asthma severity or baseline asthma control/lung function. This study therefore provides reassuring evidence to justify the use of LAIV in children with moderate to severe asthma.

Concerns with respect to LAIV in children with wheezing were first raised in 2 randomized, double-blind trials. Bergen et al^8^ reported a significantly increased relative risk (4.06; 90% CI, 1.29-17.86) for wheezing in the 42 days after vaccination in children aged 18 to 35 months with a history of “asthma or reactive airways disease,” despite the study specifically excluding children with a parent-reported history of asthma.^8^ In a second study, Belshe et al^7^ reported an increased rate of “medically significant wheezing” in infants aged 6 to 11 months receiving LAIV compared with injected influenza vaccine (IIV) (13.6% LAIV vs 10.4% IIV), but not in children older than 2 years (2.1% LAIV vs 2.5% IIV).^7^ The authors further noted a higher (but not significant) rate of hospitalization in children with a history of wheezing, although the study excluded any participant with a history of wheezing in the 42 days before LAIV.

In contrast, numerous other studies, including data from randomized controlled trials^9^, ^10^, ^11^, ^12^ and postmarketing population-based studies,^13^, ^14^, ^15^, ^16^, ^17^ have found no evidence for an adverse safety signal in young people with asthma older than 2 years (which is the lowest age that LAIV is licensed for use in). Ambrose et al undertook an analysis of 2 previously published randomized multinational trials,^7^^,^^12^ which when combined included 1940 children aged 2 to 5 years with asthma or a history of wheezing, and found no evidence for increased risk of wheezing after LAIV compared with IIV, although subjects with an episode of wheeze in the 42 days before vaccination were excluded.^28^ In the SNIFFLE-2 study, we did not identify an adverse safety signal after LAIV in 445 children with mild to moderate asthma.^19^ More recently, an analysis of data from the Clinical Practice Research Datalink relating to more than 11,000 young people at a “higher risk” for influenza disease (including patients with asthma, who constituted more than 70% of the cohort) receiving LAIV in general practice in the United Kingdom found no evidence for increased hospitalization after vaccination,^29^ although as the authors comment, allocation to treatment (LAIV, IIV, or no vaccine) was not controlled and therefore the results may have been confounded by differences between treatment groups at baseline.

Many studies have used a definition of “medically significant wheeze” (ie, wheeze that requires review by a health care professional) in the 42 days postvaccination as the outcome measure for lower respiratory tract symptoms, which is insensitive because parents of children with recurrent wheezing will often manage their child’s symptoms at home without recourse to a health care professional. These studies also frequently exclude children with wheezing up to 42 days before vaccination, and so exclude those with significant underlying disease who are likely to be at a greater risk of lower respiratory tract events after LAIV. In this study, children with active wheezing in the previous 3 days were not specifically excluded (subject to assessment by the site lead investigator) and our strategy to specifically recruit young people with severe or “difficult” asthma provide useful data on the safety of LAIV in children with more symptomatic disease.

### Strengths and limitations of study

To focus on children with more severe asthma, we specifically targeted patients attending specialist asthma clinics in the United Kingdom, in whom asthma treatment had been guided by specialists in severe asthma. To this extent, around 50% of participants met international criteria for severe or difficult asthma. Although the optimal design would have randomized participants to receive either LAIV or IIV (with appropriate placebo controls), we considered that this would have adversely affected recruitment in our target population. We therefore opted for an open, observational design rather than a placebo-controlled, more invasive study (using IIV/placebo injection) to maximize recruitment.

Despite this, recruitment to this study was challenging because more than 50% of children screened for inclusion through our severe asthma clinics had already received LAIV through the national vaccine program. This raises an issue in terms of the extent to which public health guidance filters through to those administering vaccine in the community and through primary care: under previous guidance from Public Health England, children receiving high-dose ICSs “should only be given LAIV on the advice of their specialist,” yet clearly this advice was not always adhered to.

We did not identify any evidence to suggest that wheezing or asthma exacerbation postvaccination was related to baseline severity, asthma control, or lung function, nor did we find any evidence to suggest a systematic worsening of asthma control due to LAIV. Follow-up was complete in only 67% of participants, but there were no obvious differences in baseline asthma control between those with follow-up and those without, implying that baseline asthma control was not a confounder. We excluded children with a history of an episode of invasive ventilation due to a respiratory illness in the preceding 2 years, and therefore we cannot make any recommendations for children in this category.

These data provide evidence for the safety of LAIV in children with severe asthma, despite the above limitations with respect to the absence of a control group receiving IIV or placebo and the lower-than-anticipated recruitment and lack of 4-week follow-up data in one-third of participants. To provide further reassurance, we also interrogated our national pharma-surveillance scheme for adverse events due to LAIV in children with asthma. Given the widespread use of LAIV in the UK pediatric population, including in patients with severe asthma (as evidenced by our observation that many potential participants had already been given LAIV when approached), we considered this might provide further useful data. Over a 5-year period to 2018, there were 41 notifications of asthma exacerbation post-LAIV (an average of 8 per annum), although of course these could be unrelated to LAIV. In the context of more than 3 million doses of LAIV given annually to this age group, these figures provide additional reassurance.

### Conclusions and policy implications

This study provides evidence for the revised UK guidance for the 2019/2020 season that “children with asthma on inhaled corticosteroids may safely be given LAIV, irrespective of the dose prescribed,” although LAIV continues to be not recommended in those with an acute exacerbation of asthma symptoms in the previous 72 hours.^6^

**Clinical implications**

This study provides evidence that children with asthma may safely be given LAIV irrespective of the dose of ICSs prescribed, although LAIV is not recommended in those with an acute exacerbation of asthma symptoms.

## Figures and Tables

**Table E1:** Comparison between those subjects with follow-up data relating to asthma control at 4 weeks and those without

Characteristic	Subjects with follow-up data (n = 319)	Subjects without follow-up data (n = 159)	*P* value
Age (y)			
2-4	60 (19)	25 (16)	.65
5-11	157 (49)	78 (49)	
12+	102 (32)	56 (35)	
Sex: male	190 (60)	98 (62)	.69
Baseline asthma control score, median (IQR)			
TRACK (age 2-4 y)	50 (30-65)	60 (38-75)	.20
(c)ACT (age 5-11 y)	19 (16-22)	19 (13-23)	.34
ACT (age 12+ y)	19 (17-22)	19 (14-22)	.16
ACQ (age 5+ y)	6 (2-10)	6 (2-13)	.24
Receiving high-dose ICSs			
Age 2-4 y: ICS ≥ 400 μg/d	33 (10)	15 (9)	.87
Age 5-11 y: ICS ≥ 800 μg/d	40 (13)	23 (14)	.57
Age 12+ y: ICS ≥ 800 μg/d	54 (17)	43 (27)	.01
Previous influenza vaccination	225 (71)	107 (67)	.53
Previous LAIV	156 (49)	66 (42)	.14

*IQR*, Interquartile range.

Values are n (%) unless otherwise indicated.

**Table E2:** Change in symptom control ≥minimum important difference for study population, and by severity definition

Population	n	Improved	No change	Worse	McNemar *P* value
Complete cohort					
All ages	319	77 (24)	148 (46)	94 (29)	.14
2-4 y	60	19 (32)	17 (28)	24 (40)	.54
5+ y	259	58 (22)	131 (51)	70 (27)	.18
5-11 y	157	40 (25)	76 (48)	41 (26)	.71
12+ y	102	18 (18)	55 (54)	29 (28)	.08
“Persistent poor control”					
All ages	147	42 (29)	54 (37)	51 (35)	.46
2-4 y	45	17 (38)	10 (22)	18 (40)	.95
5+ y	102	25 (25)	44 (43)	33 (32)	.32
5-11 y	56	15 (27)	25 (45)	16 (29)	.83
12+ y	46	10 (22)	19 (41)	17 (37)	.24
High-dose ICS					
All ages	127	35 (28)	50 (39)	42 (33)	.46
2-4 y	33	13 (39)	7 (21)	13 (39)	.84
5+ y	94	22 (23)	43 (46)	29 (31)	.26
5-11 y	40	12 (30)	19 (48)	9 (22)	.63
12+ y	54	10 (19)	24 (44)	20 (37)	.07
“Difficult asthma”					
All ages	90	34 (38)	31 (34)	25 (28)	.30∗
2-4 y	37	15 (41)	9 (24)	13 (35)	.58
5+ y	53	19 (36)	22 (42)	12 (23)	.23∗
5-11 y	24	10 (42)	9 (38)	5 (21)	.21∗
12+ y	29	9 (31)	13 (45)	7 (24)	.62∗

Values are n (%).

∗ This severity definition is partially dependent on the (c)ACT score, so this *P* value may be influenced by regression to the mean.

**Table E3:** AEFI reported within 2 h of LAIV administration

No.	Time to symptoms (min)	Description of events	IDMC review: possible allergic etiology?	Causality (due to LAIV)
1	20-60	Mild itchy facial rash; no recurrence with second dose of LAIV given 1 mo later	Yes	Unlikely
2	5-20	Mild generalized pruritus, no rash ?due to environmental exposure	Yes	Possible
3	5-20	Itchy facial rash ?contact reaction from vaccine	Yes	Probable
4	20-60	Rhinitis, facial hives, and mild angioedema. Possible causation due to exposure to a food allergen	Yes	Unlikely
5	5-20	Localized urticaria to nasal bridge and mild upper-lip swelling ?contact reaction from vaccine	Yes	Probable
6	5-20	Nausea	No	
7	5-20	Itchy nose	No	
8	20-60	Occasional sniffing	No	
9	20-60	Generalized mild rash and itchy throat. No other symptoms. Frequently develops similar symptoms due to environmental exposure	Yes	Unlikely

*AEFI*, Adverse event following immunization.

**Table E4:** Relationship between the occurrence of delayed events (in the 72 hours after LAIV) and baseline characteristics

Factor	Level	All delayed AEs	*P* value	Lower respiratory tract AEs only	*P* value
Age	Median, IQR	8.7 (5.0-12.3)	.02	7.8 (4.2-11.9)	.01
	Age 2-4 y	35 of 77 (45%)		18 of 77 (23%)	
	Age 5-11 y	65 of 220 (30%)		24 of 220 (11%)	
	Age 12+ y	39 of 143 (27%)		14 of 143 (10%)	
Baseline (c)ACT or TRACK score	Q1∗	34 of 92 (37%)	.12†	12 of 92 (13%)	.77†
	Q2∗	33 of 97 (34%)		15 of 97 (15%)	
	Q3∗	44 of 145 (30%)		17 of 145 (12%)	
	Q4∗	28 of 103 (27%)		12 of 103 (12%)	
Lung function (FEV_1_ or PEFR)	>95%	30 of 106 (28%)	.59†	12 of 106 (11%)	.97†
	95%-90%	12 of 38 (32%)		4 of 38 (11%)	
	89%-90%	14 of 55 (25%)		3 of 55 (5%)	
	79%-70%	9 of 36 (25%)		6 of 36 (17%)	
	69%-60%	7 of 20 (35%)		1 of 20 (5%)	
	59%-50%	4 of 10 (40%)		2 of 10 (20%)	
	<50%	1 of 2 (50%)		0 of 2 (0%)	
Baseline ACQ score	0-4	41 of 160 (26%)	.22†	14 of 160 (9%)	.23†
	5-9	28 of 94 (30%)		10 of 94 (11%)	
	10-14	20 of 58 (34%)		11 of 58 (19%)	
	15-19	6 of 30 (20%)		1 of 30 (3%)	
	20-24	8 of 17 (47%)		2 of 17 (12%)	
	25-30	1 of 3 (33%)		0 of 3 (0%)	
“Persistent poor control”	No	70 of 235 (30%)	.41	26 of 235 (11%)	.31
	Yes	69 of 205 (34%)		30 of 205 (15%)	
High-dose ICS	No	73 of 257 (28%)	.10	28 of 257 (11%)	.19
	Yes	66 of 183 (36%)		28 of 183 (15%)	
“Difficult asthma”	No	97 of 316 (31%)	.57	35 of 316 (11%)	.11
	Yes	42 of 124 (34%)		21 of 124 (17%)	
Atopic status					
Eczema	No	64 of 224 (29%)	.18	31 of 224 (14%)	.57
	Yes	75 of 216 (35%)		25 of 216 (12%)	
Allergic rhinitis	No	46 of 168 (27%)	.14	21 of 168 (13%)	1.0
	Yes	93 of 272 (34%)		35 of 272 (13%)	
IgE-mediated food allergy	No	79 of 239 (33%)	.54	34 of 239 (14%)	.32
	Yes	60 of 201 (30%)		22 of 201 (11%)	
Previous influenza vaccine	No	42 of 119 (35%)	.42	13 of 119 (11%)	.63
	Yes	96 of 308 (31%)		41 of 308 (13%)	

*PEFR*, Peak expiratory flow rate.

Four children met the criteria for severe exacerbation within 72 hours, aged 3, 4, 7, and 15 years; 3 had coexisting atopy, and 2 (aged 3 and 7 years) had previously received LAIV.

∗ Data analyzed in quartiles, for ACT this was <15,15-18,19-22, and ≥23; for TRACK, <35, 35-50, 55-65, and ≥70.

† Trend test across categories.

**Table E5:** Serious AEs reported during the study

No.	Description of event	Causal relationship	Expected (Y/N)
1	Subject with severe asthma developed viral symptoms and wheeze 1 wk post-LAIV. Assessed in hospital: oxygen saturations 94% in room air. Admitted for 3 d observation, no change in medical management instituted	Probable	Y
2	Subject with severe asthma noted to have possible early signs of chest infection noted at assessment pre-LAIV, and oral antibiotics commenced. LAIV administered after review by local Investigator. Three days later, subject presented to local hospital with increased wheeze and breathlessness, and admitted for 3 d. Treated with nebulized salbutamol and intravenous antibiotics	Possible	Y
3	Subject with severe asthma; 19 d after LAIV, asthma exacerbation and was admitted to hospital overnight for hourly nebulizers, magnesium sulfate, and intravenous antibiotics. Discharged the next day	Unlikely∗	Y
4	Subject has severe asthma, previous HDU admissions, tolerated LAIV in previous years. Three weeks after LAIV, developed increasing wheeze/cough, prompting presentation to hospital on day 3 of illness. Diagnosed with asthma exacerbation and admitted to HDU, given magnesium sulphate, aminophylline, steroids, and nebulized salbutamol. No antibiotics started. No virology sent, blood studies demonstrated neutrophilia with elevated CRP (48 mg/L). No focal abnormality on chest x-ray. Discharged home after 48 h	Unlikely∗	Y

*CRP*, C-reactive protein; *HDU*, high dependency unit; *IDMC*, Independent Data Monitoring Committee; *N*, no; *PI*, principal investigator; *Y*, yes.

∗ IDMC ascribed unlikely due to very low probability of ongoing viral shedding 3 weeks after LAIV.^E1^
